# Authors’ Reply: Toward Retrieval-Grounded Evaluation for Conversational Large Language Model–Based Risk Assessment

**DOI:** 10.2196/91981

**Published:** 2026-03-12

**Authors:** Mohammad Amin Roshani, Xiangyu Zhou, Yao Qiang, Srinivasan Suresh, Steve Hicks, Usha Sethuraman, Dongxiao Zhu

**Affiliations:** 1 Department of Computer Science, Wayne State University, 5057 Woodward Ave, Detroit, MI, 48202, United States, 1 3135773104; 2 Oakland University, Rochester, MI, United States; 3 UPMC Children's Hospital of Pittsburgh, Pittsburgh, PA, United States; 4 Department of Pediatrics, Pennsylvania State University, Hershey, PA, United States; 5 Children's Hospital of Michigan, Detroit, MI, United States

**Keywords:** personalized risk assessment, large language model, artificial intelligence, conversational AI, COVID-19

 We thank the authors of the letter to the editor [1] for their thoughtful perspective and for highlighting the potential value of retrieval-grounded sensitivity analyses alongside conventional area under the receiver operating characteristic curve (AUC) reporting. We welcome this opportunity to further clarify the design choices and evaluation scope of our published work.

As described in Roshani et al [2], the system explicitly provides 2 distinct user-facing interfaces: a clinician-facing interface and a patient-facing interface (these are shown in Figure 3 in Roshani et al [2]), each designed for a different purpose. We agree that subgroup-level audits are essential; accordingly, our clinician-facing interface already supports stratified performance review (eg, AUC by demographic variables), laying the groundwork for more systematic fairness analyses in future versions.

In contrast, the patient-facing interface prioritizes interpretability and usability rather than aggregate performance metrics. It presents both a binary classification (severe vs nonsevere) and an individualized continuous risk score derived from the model’s logit output, where higher values indicate greater severity. This output is further complemented by attention-based feature importance to support transparent, conversational risk assessment. Notably, the patient-facing interface produces no free text in clinical language, so the risk of hallucinated clinical statements is not applicable to our system’s current design.

Regarding retrieval grounding, the letter raises an important direction for future evaluation. We note that retrieval-based augmentation requires the availability of stable, domain-specific corpora. In the published study, our primary objective was to investigate the feasibility and performance of generative white-box large language models (LLMs) in low-data, emergent-disease settings. As such, we primarily considered a novel infection scenario, in which trusted external knowledge sources may be sparse, incomplete, or evolving. This was the case for pediatric COVID-19 during its early emergence, when authoritative, age-specific guidelines were limited, making LLM-only approaches more practical at the time. In this context, the system is intended to provide an initial, clinician-informed risk assessment, leveraging a small number of curated cases incorporated during fine-tuning, rather than relying on external retrieval. We therefore presented the reported system as an initial in-house baseline, designed to function in early-stage or data-limited settings.

As clinical knowledge bases mature, the same conversational pipeline can naturally be extended to incorporate explicit evidence grounding and source citation, enabling more personalized and evidence-supported risk assessment. We appreciate the authors’ comments in highlighting this direction and view it as complementary to, rather than in conflict with, the scope and objectives of the published work.

## Abbreviations

AUC: area under the receiver operating characteristic curve
LLM: large language model
